# Monitoring of endoscope reprocessing with an adenosine triphosphate (ATP) bioluminescence method

**DOI:** 10.3205/dgkh000289

**Published:** 2017-03-27

**Authors:** Nina Parohl, Doris Stiefenhöfer, Sabine Heiligtag, Henning Reuter, Dana Dopadlik, Frank Mosel, Guido Gerken, Alexander Dechêne, Evelyn Heintschel von Heinegg, Christoph Jochum, Jan Buer, Walter Popp

**Affiliations:** 1 HyKoMed GmbH, Dortmund, Germany; 2 Department of Gastroenterology and Hepatology, University Hospital Essen, Germany; 3 3M Deutschland GmbH, Neuss, Germany; 4 Department of Hospital Hygiene, University Hospital Essen, Germany; 5 Department of Clinical Microbiology, University Hospital Essen, Germany; 6 HyKoMed, Dortmund, Germany

**Keywords:** endoscope reprocessing, contamination, process control, surveillance, ATP, adenosine triphosphate, microbial cultures

## Abstract

**Background:** The arising challenges over endoscope reprocessing quality proposes to look for possibilities to measure and control the process of endoscope reprocessing.

**Aim:** The goal of this study was to evaluate the feasibility of monitoring endoscope reprocessing with an adenosine triphosphate (ATP) based bioluminescence system.

**Methods:** 60 samples of eight gastroscopes have been assessed from routine clinical use in a major university hospital in Germany. Endoscopes have been assessed with an ATP system and microbial cultures at different timepoints during the reprocessing.

**Findings:** After the bedside flush the mean ATP level in relative light units (RLU) was 19,437 RLU, after the manual cleaning 667 RLU and after the automated endoscope reprocessor (AER) 227 RLU. After the manual cleaning the mean total viable count (TVC) per endoscope was 15.3 CFU/10 ml, and after the AER 5.7 CFU/10 ml. Our results show that there are reprocessing cycles which are not able to clean a patient used endoscope.

**Conclusion:** Our data suggest that monitoring of flexible endoscope with ATP can identify a number of different influence factors, like the endoscope condition and the endoscopic procedure, or especially the quality of the bedside flush and manual cleaning before the AER. More process control is one option to identify and improve influence factors to finally increase the overall reprocessing quality, best of all by different methods. ATP measurement seems to be a valid technique that allows an immediate repeat of the manual cleaning if the ATP results after manual cleaning exceed the established cutoff of 200 RLU.

## Zusammenfassung

**Hintergrund:** Die zunehmenden Anforderungen an die Qualität der Endoskopaufbereitung führen dazu, nach neuen Möglichkeiten der Endoskopüberwachung sowie der Prozesskontrolle zu suchen.

**Ziel:** Ziel dieser Studie war es, die Möglichkeit der Überwachung der Endoskop-Aufbereitung mit einem Adenosintriphosphat (ATP)-basierten Biolumineszenz-System zu bewerten.

**Methoden:** 60 Proben von acht unterschiedlichen Gastroskopen wurden im Rahmen der routinemäßigen klinischen Anwendung an einem großen Universitätsklinikum in Deutschland untersucht. Die Endoskope wurden mittels ATP-Messung und mikrobiologischer Kontrollen (Spülproben) zu unterschiedlichen Zeitpunkten des Aufbereitungsprozesses beurteilt.

**Befunde:** Nach der Vorreinigung lag der mittlere ATP-Wert bei 19.437 relativen Lichteinheiten (RLU), nach der Bürstenreinigung bei 667 RLU und nach dem Reinigungs- und Desinfektionsgerät für Endoskope (RDG-E) bei 227 RLU. Nach der Bürstenreinigung betrug die mittlere Anzahl koloniebildender Einheiten (KBE) pro Endoskop 15,3 KbE / 10 ml und nach der Aufbereitung im RDG-E 5,7 KbE / 10 ml. Unsere Ergebnisse zeigen, dass es Aufbereitungszyklen gibt, die nicht zur vollständigen Reinigung eines Endoskops führen, das am Patienten eingesetzt wurde.

**Fazit:** Die Ergebnisse zeigen, dass die Überwachung flexibler Endoskope mit der ATP-Messung eine Reihe unterschiedlicher Einflussfaktoren wie den Endoskopzustand und die Art des endoskopischen Eingriffs (invasiv/non-invasiv) sowie insbesondere die Qualität der Vorreinigung und der Bürstenreinigung vor der Aufbereitung im RDG-E identifizieren kann. Eine erweiterte Prozesskontrolle, möglichst durch verschiedene Methoden, stellt eine Möglichkeit dar, um Einflussfaktoren auf die Aufbereitungsqualität zu identifizieren und zu verbessern und damit die gesamte Endoskopaufbereitung zu verbessern. Die ATP-Messung erscheint uns als zuverlässige Technik, die eine sofortige Wiederholung der manuellen Reinigung ermöglicht, wenn die ATP-Ergebnisse nach der manuellen Reinigung den vorgeschlagenen Grenzwert von 200 RLU überschreiten.

## Introduction

Since 2000 several countries have implemented guidelines on the reprocessing of endoscopes, but in many countries guidelines are still not available. In Germany health care providers who are reprocessing endoscopes are legally required to follow the guidelines of the “committee of hospital hygiene and infection control” (KRINKO) [1]. This development has improved the reprocessing of flexible endoscopes over the last couple of years, but past literature reviews have still shown an infection risk by patient to patient transmission during endoscopic procedures [2], [3], [4], [5], [6]. Authors agree that the reported figures may be biased by underreporting complications, infections and outbreaks as patients are usually discharged soon after the procedure.

Recently the number of publications on outbreaks associated with endoscopes has increased tremendously [7], [8], [9], [10], [11]. All of these outbreaks have only been identified as they were associated with multidrug-resistant gram-negative (MDRGN) bacteria and these outbreaks have been identified by routine hospital screening programs and would have been missed under other circumstances. 

This development shows that the decontamination of endoscopes continues to be challenging [3], [12], [13], [14]. In the past all endoscope associated outbreaks have been connected to breaches in the endoscope decontamination process and also to damaged channels [9]. However, newer outbreaks suggest that the complicated design of the endoscopes may result in inappropriate reprocessing outcome even if the decontamination process has been strictly followed [7].

The narrow lumen, lumen transitions to stainless steel parts, and valves are difficult to access for cleaning and decontamination. Visual control of the decontamination process is hardly possible. Next to the difficulties caused by the device construction, a majority of decontamination process parameters, like endoscope and equipment condition, defects and decontamination chemistry, have a strong impact on the outcome of the decontamination success. Additionally many process steps are manually performed by the operator and therefore heavily depend on the training, education, skill and general compliance of the individual operator and are difficult to validate. 

This situation proposes to look for possibilities to better measure and control the process of endoscope reprocessing. The reprocessing and sterilization of surgical instruments is a closely monitored process. In contrast to that, the reprocessing of flexible endoscopes mainly relies on training of the operators and the documentation of the validated AERs.

The goal of this study was to evaluate the feasibility of an routine endoscope-specific monitoring of the reprocessing with ATP based bioluminescence system. The study should

- assess the capability of ATP as a process control marker,
- assess the correlation between ATP measurement at different process steps,
- the correlation between ATP and microbial contamination and
- assess the overall process quality at a German hospital.

## Methods

Gastroscopes from daily routine have been tested at different steps during routine reprocessing. Eight gastroscopes from the same manufacturer and similar endoscope designs were defined as study endoscopes. 

The reprocessing followed the German KRINKO guidelines [1] on the reprocessing of thermolabile endoscopes and consisted of bedside flushing of the endoscope, manual cleaning of the endoscope and consecutive cleaning and disinfection in an automated endoscope reprocessor (AER). Two dedicated, validated AERs (Olympus ETD 3) with recommended cleaning detergents (Olympus Cleaner) and disinfectant (Olympus Disinfectant, glutaraldyhde) were used in this study. Manual cleaning has been performed with common, reusable brushes and an enzymatic detergent (Bodedex Forte). Channels have been brushed twice or until no debris was detected on the brush. All endoscopes in this study have been processed by a single operator.

The gastroscopes have been sampled after the bedside flush, after the manual cleaning and after the AER. According to a previously reported sample process [15], [16] 40 ml of sterile water was flushed through the biopsy/suction channel from the umbilical end to the distal end of the endoscope. Afterwards 60 ml of air was used to collect residual sample water inside the endoscope. 10 ml of the sample has been used to measure the ATP level in triplicate and the remaining sample volume has been used for the microbial analysis. Sterile sampling water supply was tested for ATP level for each individual endoscope in triplicate. 

3M Clean Trace NGI bioluminometer and 3M Clean Trace ATP Water tests have been used to measure the ATP level of the sample in relative light units (RLU).

Microbiological evaluation has been performed according to established standard operating procedures at the Institute of Clinical Microbiology at the University Clinic Essen [17], [18]. The total viable count has been done by filtration of 10 ml sample fluid through a membrane filter (0.45 µm) and consecutive incubation of the filter at 36±1°C for 44±4 h on Columbia sheep blood agar for total bacterial count. Colony count was performed with a magnifying colony counter. All samples have been tested for microbial inhibitory substances. 

The study was conducted between October and December 2014 on six non-consecutive days at the University Hospital Essen, Germany. Testing of endoscopes was continued until 25 endoscopes with an RLU<200 and 25 endoscopes with an RLU>200 after manual cleaning were identified [15], [16]. Finally 60 endoscopes (27 endoscopes <200 RLU, 33 endoscopes >200 RLU) were tested to reach that goal.

All statistical analysis was done with Minitab 17.1.0. Descriptive statistics is presented for original data and log-transformed data. ANOVAs and process control analysis are done with log-transformed data only.

## Results

An overview of all results is summarized in Table 1 (Tab. 1).

The mean ATP value of the sterile water flush solution was 7.8 RLU (N=173; SD: 6.0 RLU, median: 6.0 RLU). Seven measurements are missing.

After the bedside flush the mean ATP in relative light units (RLU) was 19,437 RLU (N=180; SD: 29419 RLU, median: 8,096 RLU), after the manual cleaning 667 RLU (N=176; SD: 752 RLU, median: 225 RLU) and after the AER 227 RLU (N=180; SD: 250 RLU, median 128 RLU). The log transformed values are 3.87 (SD: 0.65, median: 3.9), 2.44 (SD: 0.65, median: 2.4) and 2.15 (SD: 0.43, median: 2.1) accordingly. For one endoscope all three repeats of the post manual ATP measurements are missing. For another endoscope one repeat of a post manual ATP measurement is missing.

An ANOVA of the log RLU shows a significant difference (p<0.001) of the different measurement points after bedside flush (mean: 3.87, 95% CI: 3.78; 3.97), after manual cleaning (mean: 2.44, 95% CI: 2.34; 2.54) and after the AER (mean: 2.15, 95% CI: 2.09; 2.21). A Pearson correlation of the log RLU of the after bedside flush and post manual cleaning shows a correlation coefficient of 0.75 (p<0.001) and between post manual cleaning and after the AER a correlation coefficient of 0.71 (p<0.001).

After the manual cleaning the mean total viable count (TVC) per endoscope was 15.3 CFU/10 ml (N=56; SD: 43.3 CFU/10 ml; median: 1.5 CFU/10 ml), and after the AER 5.7 CFU/10 ml (N=58; SD: 11.2 CFU/10 ml; median: 1.0 CFU/10 ml). A Box Cox transformation of the data with an offset of one and a consecutive two-sample t-test showed no statistical significance (p=0.09). Four endoscopes for the post manual analysis and two endoscopes for the post AER analysis showed uncountable CFUs on the microbiology report and were excluded from the analysis.

**Figure 1:**
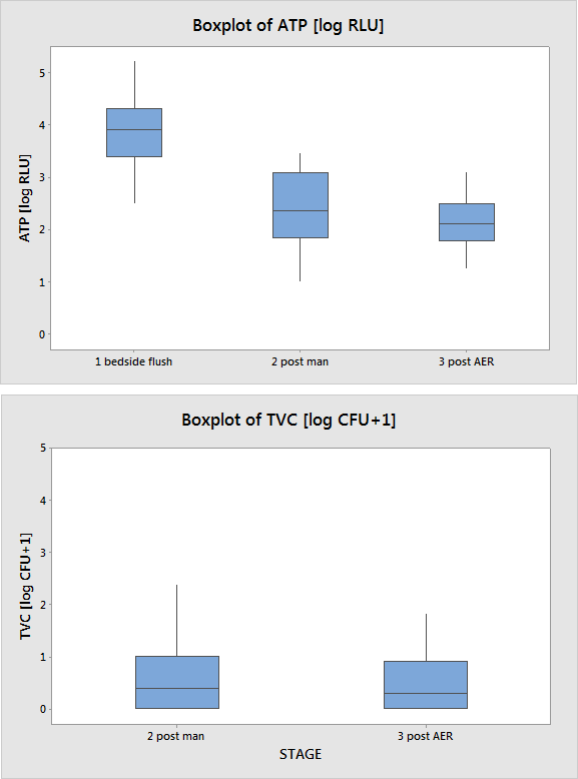
Boxplots of ATP [log RLU] and TVC [log CFU+1*] per time point * An offset of one has been added to all CFU counts to be able to perform a log transformation for the statistical analysis.

Boxplots of the log RLU and TVCs are given in Figure 1 (Fig. 1).

**Figure 2:**
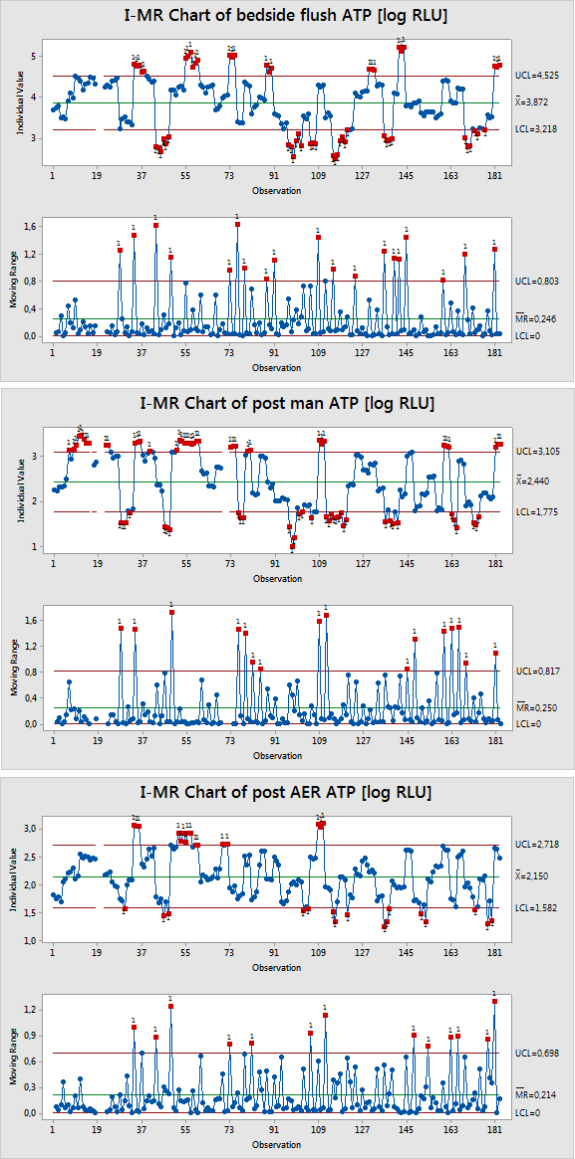
Control charts of log RLU after bedside flush, after manual cleaning and after AER

**Figure 3:**
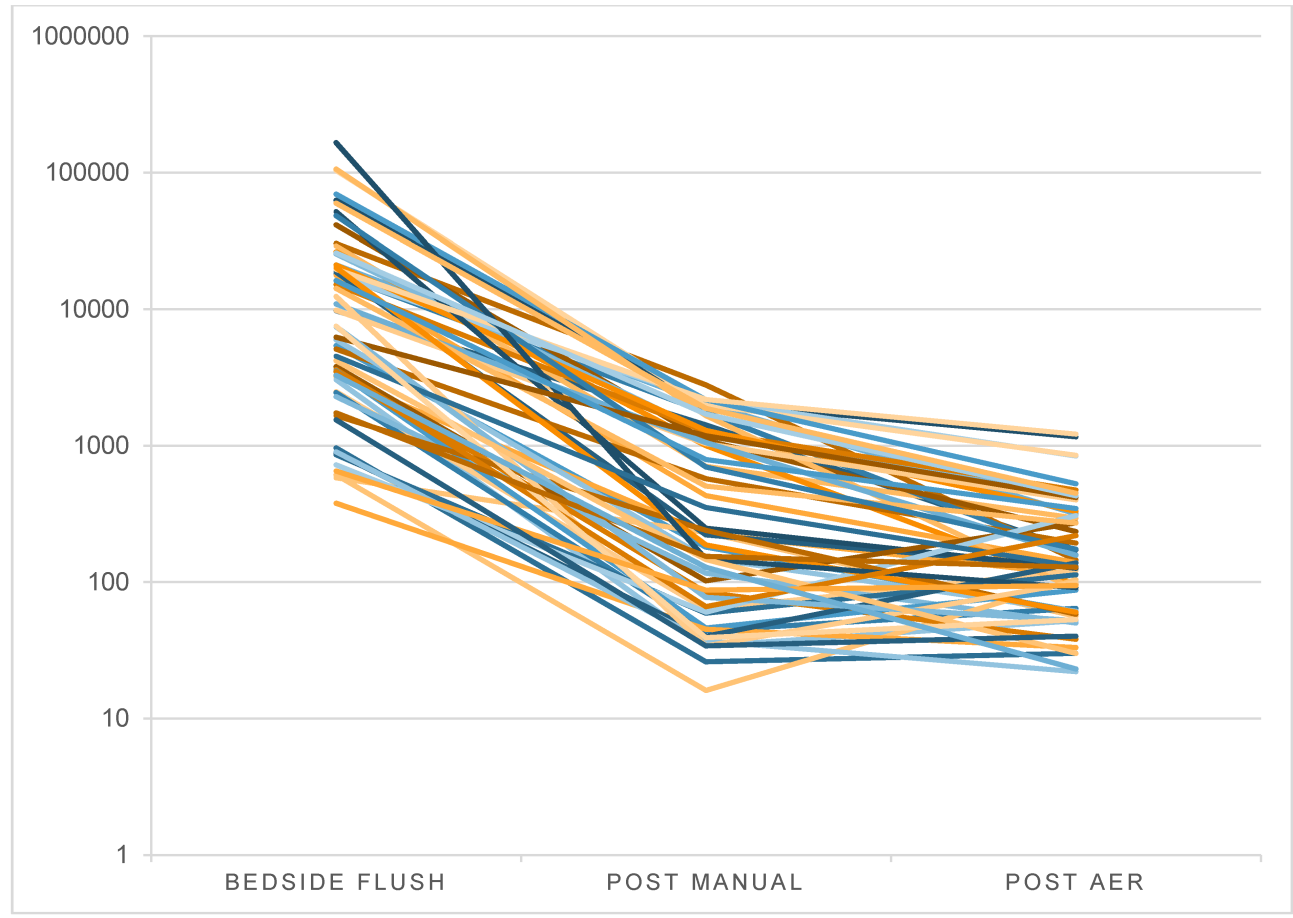
ATP level of all endoscopes per process step [log median RLU]; each line represents a single endoscope during a full reprocessing cycle.

For a process control analysis statistical control charts of ATP measurements at the different process steps as log RLU are shown in Figure 2 (Fig. 2). These charts give the level and variance of the given process step and highlight endoscopes that fall outside a calculated process variability, which is defined by the upper and lower control limits. The control limits are not necessarily clinically relevant, but each measurement outside the control limits represents an event that occurred in this endoscopic procedure or reprocessing cycle that caused this cycle to be outside the normal cleaning process distribution, meaning this endoscope is cleaner or dirtier than the average endoscope. The calculated upper control limits after bedside flush, after manual cleaning or after the AER are 4.53, 3.10 and 2.72 accordingly. The lower control limits are 3.22, 1.78 and 1.58. The data confirm that the observed process has a relatively high variance. Figure 3 (Fig. 3) shows the overall RLU decrease over the different process steps for all 60 study endoscopes. An endoscope that enters the reprocessing procedure at a lower contamination level after bedside flush continues to maintain a low level of contamination through the process. This observation is confirmed by the high correlation coefficient of 0.71.

## Discussion

At the moment, process control in reprocessing of flexible endoscopes is usually done by microbiologic methods. These methods are relatively complex and therefore are usually done on a low frequency (monthly, quarterly, yearly) and that results are available only after some days and in the meantime the endoscope may be used on a lot of patients. Therefore, this method is not promising for a more frequent monitoring and alternatives are urgently needed. Protein testing and ATP measurement are two of those and ATP measurement is interesting because it is easy to handle, shows results after seconds and can enable instant decisions whether to reprocess again or not [8], [19]. It seems that the ATP test is more sensitive than protein or blood tests in controlling cleaning of endoscopes [14].

Our study included measurement of ATP in endoscopy channels after bedside flush, after manual cleaning and after washer disinfector (AER) that means before use at the next patient. We could show a decrease in mean log transformed ATP levels from 3.87 after bedside flush to 2.44 after manual cleaning and 2.15 after the AER. The mean ATP level after bedside flush and after manual cleaning in our study are very similar to the result of previously reported studies [8], [14], [20]. These data underline that manual cleaning is very effective and necessary.

Also there was a correlation of ATP levels between each step. That means that a higher contamination at the beginning usually is ending with a higher contamination at the end of the process. This was also found in the study of Visrodia et al. (2014) [20].

A surprising result is that some endoscopes showed a higher ATP level at the end of disinfection than after manual cleaning. This means that some of the endoscopes get dirtier during the final AER process or more contamination become visible. The reason for that is not clear and need further investigation. 

We saw a slight decrease of mean CFU counts in the flush samples after manual cleaning and after AER. This decrease was not significant and no correlation has been observed. If this marginal decrease is caused by a compromised condition of the used AER or by other factors like cleaning performance is difficult to say as no correlation of the observed CFU data occurred. We also could not see a direct correlation between ATP measurements and CFU counts. However, a correlation would have been unexpected at the low CFU counts after manual cleaning or after the AER [15], [21], [22].

If we split the tested endoscopes into a “clean” and a “dirty” group, according to the established cutoff of 200 RLU after manual cleaning [15], [16], we can compare those groups in regards to the microbial contamination after the AER. This would give us a prediction of the final reprocessing outcome after the AER, based on a cleaning test after the manual cleaning.

In Germany a reprocessed endoscope is allowed to carry 10 CFU/10 ml of viable bacteria [17]. In the group of endoscopes that have an ATP level above 200 (N=31), seven endoscopes had a total viable count above 10 CFU/10 ml. In the group below 200 RLU (N=26), only two endoscopes had a total viable count above 10 CFU/10 ml. 

In an analysis of influence factors on the reprocessing outcome, we saw a clear influence of invasive procedures in our study (p<0.001), which leaves a bigger risk of insufficient cleaning result for those procedures. There were also hints for an influence of the endoscope on the reprocessing outcome. The endoscope influence was not correlated with the age of the endoscope and may be a result of the general condition of the endoscope. 

Basically our results show that the reprocessing of flexible endoscopes is showing a high process variability and some cycles are not able to clean a patient used endoscope which is in accordance with the report of Ofstead et al. [14]. Our data suggest that this is driven by a number of different influence factors that include endoscope condition, the endoscopic procedure itself and especially the quality of the bedside flush and manual cleaning before the AER. This last finding is supported by a lot of outbreak reports in the last years [2], [3], [4], [5], [7], [10], [11]. Although all AER manufacturers correctly require to perform manual cleaning before the AER process, the operators often trust in the validated AERs to manage all manual cleaning lapses and have developed a false sense of safety. This is a concerning trend.

Our study did not systematically looked into all potential influence factors of endoscope reprocessing, but the presented data suggest that a closer routine monitoring of the reprocessing can identify and help eliminating potential risks in endoscope reprocessing to finally improve the reprocessing quality of flexible endoscopes. Further studies to confirm this hypothesis are required. 

At the moment more frequent process control should be done to mitigate the high process variability in endoscope reprocessing and to improve the overall reprocessing quality, best of all by different methods. 

Our data show that ATP measurement – at one or different steps, done at any reprocessing cycle or at least, e.g., once a day – might be one method to control the process. The big advantage of the method is that it is done fast and results are obtained on-site so that instant conclusions can be drawn.

## Notes

### Competing interests

The study was financially supported by 3M Germany, Neuss, Germany. Two authors are employed by 3M Germany, Neuss, Germany.

## Figures and Tables

**Table 1:**
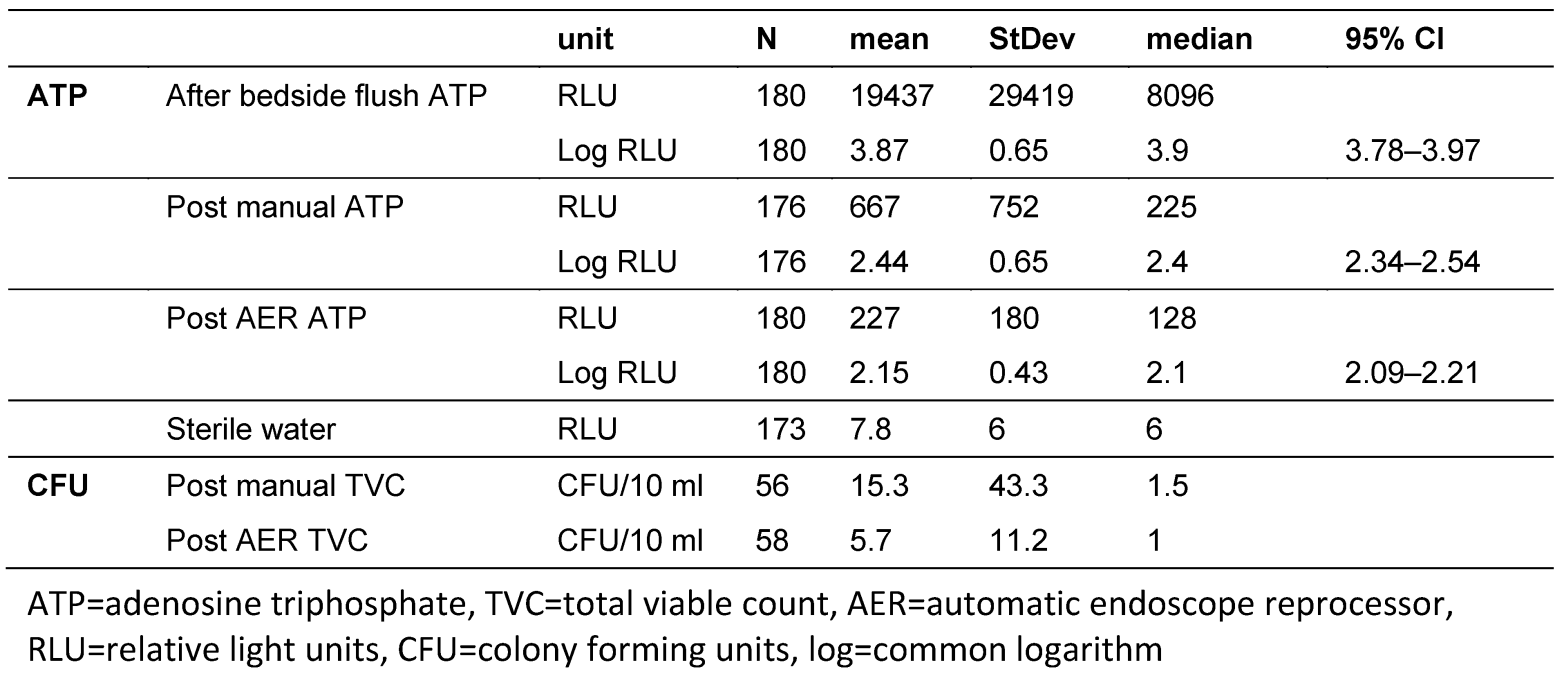
Overview of results
